# Clinical, immunological, treatment characteristics, and outcomes in 22 patients with major histocompatibility complex class II deficiency

**DOI:** 10.3389/fimmu.2026.1811502

**Published:** 2026-04-22

**Authors:** Sule Haskologlu, Caner Aytekin, Candan Islamoglu, Sevgi Kostel Bal, Kubra Baskın, Baran Erman, Tanıl Kendirli, Serdar Ceylaner, Figen Dogu, Aydan Ikinciogullari

**Affiliations:** 1 Department of Pediatric Immunology and Allergy, and Hematopoietic Stem Cell Transplantation Unit, Faculty of Medicine, Ankara University, Ankara, Türkiye; 2 Department of Pediatric Allergy and Immunology, Dr. Sami Ulus Children’s Health and Diseases Training and Research Hospital, Ankara, Türkiye; 3 Department of Pediatric Immunology and Allergy, Gülhane Training and Research Hospital, Ankara, Türkiye; 4 Department of Pediatric Immunology and Allergy, Faculty of Medicine, Gazi University, Ankara, Türkiye; 5 Can Sucak Research Laboratory for Translational Immunology, Hacettepe University, Ankara, Türkiye; 6 Department of Pediatric İntensive Care Unit, Faculty of Medicine, Ankara University, Ankara, Türkiye; 7 Intergen Genetics and Rare Diseases Diagnosis Research & Application Center, Ankara, Türkiye

**Keywords:** CD4+ T cell lymphopenia, HLA-DR, hematopoietic stem cell transplantation, MHC class II deficiency, long-term outcome

## Abstract

**Background:**

Major histocompatibility complex (MHC) class II deficiency is a rare, life-threatening primary immunodeficiency that presents in early infancy with a SCID phenotype. However, emerging data indicate substantial clinical and immunological heterogeneity, including atypical presentations and neurological involvement.

**Methods:**

We retrospectively evaluated the clinical, immunological, genetic, and treatment-related characteristics and outcomes of 22 patients from 19 unrelated families diagnosed with MHC class II deficiency at a single referral center (2000–2019). Ten patients underwent HSCT at our center; transplant-related outcomes were evaluated, and long-term follow-up data were available for the six surviving patients through 2025.

**Results:**

The median age at symptom onset and diagnosis was 9 and 12 months, respectively. Pneumonia, chronic diarrhea, and failure to thrive were the most common presenting features; neurological manifestations and developmental delay were observed in a subset of patients. Two patients showed residual HLA-DR expression and survived with a milder clinical course. CD4^+^ T cell lymphopenia and humoral dysfunction were consistent, while total lymphocyte counts were variable. RTE levels were mildly to moderately reduced in all tested patients, suggesting impaired thymic output. Severe viral infections were frequent and could be rapidly fatal. Fourteen patients required PICU admission, associated with high mortality. Genetic analysis (n:15) identified homozygous pathogenic variants in *RFXANK* (n=5), *RFXAP* (n=4), *RFX5* (n=3), and *CIITA* (n=3). Ten patients underwent HSCT, with superior survival compared to non-transplanted patients (60% vs. 18%). Among transplanted patients, survival appeared higher following RTC than MAC (75% vs. 50%). Post-transplant mortality was observed in association with severe pre-transplant disease, delayed diagnosis, graft failure, and infectious complications. At ≥10 years post-HSCT, all survivors were IVIG-independent; CD4^+^ T cell recovery was higher after MAC than RTC, while T-cell chimerism remained mixed in both groups.

**Conclusion:**

MHC class II deficiency is a SCID-like pediatric immunological emergency that is fatal without HSCT in most patients. Early diagnosis is critical, as initial infections may be rapidly progressive. HSCT provides durable engraftment and sustained clinical stability in long-term survivors. Incorporating HLA-DR expression analysis into first-line immunological screening, even in the absence of profound lymphopenia, may facilitate earlier diagnosis, prompt HSCT referral, and improve survival.

## Introduction

Major histocompatibility complex (MHC) class II deficiency, also known as bare lymphocyte syndrome type II (BLS II), is a rare autosomal recessive combined immunodeficiency (CID) caused by absent or markedly reduced expression of HLA class II molecules (HLA-DR, -DP, -DQ) on thymic epithelial cells, and antigen-presenting cells (APCs). HLA class II expression can also be induced in other cell types, including activated T cells (1, 2). MHC class II molecules are essential for antigen-specific activation of CD4^+^ T helper lymphocytes, positive and negative selection of CD4^+^ T cells during thymic development, and effective antigen presentation by B cells, thereby supporting immunoglobulin production (1, 2). Loss of MHC class II expression results in profound impairment of CD4^+^ T cell development and function, leading to combined defects in cellular and humoral immunity.

MHC class II deficiency is caused by defects in trans-acting regulatory factors required for MHC class II gene transcription rather than in the structural HLA genes themselves (2). Biallelic pathogenic variants in four genes; class II transactivator (*CIITA*), regulatory factor X-associated ankyrin-containing protein (*RFXANK*), regulatory factor X 5 (*RFX5*), and regulatory factor X-associated protein (*RFXAP*) account for all known cases, corresponding to the historical complementation groups A-D. These genes form the CIITA/RFX enhanceosome complex, and disruption of this complex abolishes coordinated transcription of MHC class II loci, leading to absent expression on APCs and thymic epithelial cells (1–5).

Most infants present with a severe combined immunodeficieny (SCID)-like phenotype characterized by recurrent respiratory and gastrointestinal infections, chronic diarrhea, failure to thrive, and high early morbidity and mortality (4–19). Opportunistic infections are common, predominantly severe viral infections particularly cytomegalovirus (CMV) along with *Pneumocystis jirovecii*, *Cryptosporidium* spp., severe bacterial pneumonias, and invasive fungal infections. Although most patients are diagnosed in early infancy, a small subset may be diagnosed later in life, occasionally through family screening or atypical presentation (14, 20–24). Less common manifestations include hepatic involvement, autoimmune cytopenias, polyarthritis, hemophagocytic lymphohistiocytosis, and neurological involvement such as ataxia and dysarthria, highlighting a broader clinical spectrum than initially appreciated (4, 7, 15, 22–24).

Despite previous reports, the clinical spectrum of MHC class II deficiency remains incompletely defined, particularly with respect to atypical and milder phenotypes and long-term outcomes following hematopoietic stem cell transplantation (HSCT).

Although initially described in North Africa, MHC class II deficiency has since been reported worldwide, particularly in populations with high rates of consanguinity, including Saudi Arabia, Kuwait, Iran and Türkiye (6, 10, 14, 15, 18, 19). To our knowledge, this study represents the largest single-center cohort of MHC class II deficiency reported from Türkiye, providing comprehensive clinical, immunological, genetic, and longitudinal follow-up data. Notably, all patients in this cohort are of Turkish origin, representing a relatively homogeneous population compared to previously reported series, which are often enriched for North African populations.

MHC class II deficiency is characterized by absent or markedly reduced HLA-DR expression on APCs and profound impairment of CD4^+^ T cell immunity. Diagnosis is typically established by flow cytometry and confirmed by genetic testing (1–5, 13, 14).MHC class II deficiency is a profound CID associated with a high risk of life-threatening infections, despite immunoglobulin replacement therapy (IgRT) and antimicrobial prophylaxis. Hematopoietic stem cell transplantation (HSCT) remains the only curative treatment; however, outcomes are often suboptimal when performed late and/or during active infection, with ongoing risks of graft failure, graft-versus-host disease (GvHD), and incomplete CD4^+^ T cell reconstitution (25–30). Without HSCT, survival is usually limited to early childhood (median 4–5 years) (6, 7, 10, 13, 14, 17, 18, 25–30).

Here, we report a cohort of 22 patients from 19 unrelated families, representing a large, single-center series with long-term follow-up. By characterizing clinical, immunological, genetic, and treatment-related features, our study expands the phenotypic spectrum of MHC class II deficiency and provides important insights into disease heterogeneity and long-term outcomes.

## Materials and methods

### Patient data

We retrospectively evaluated 22 patients from 19 unrelated families diagnosed with MHC class II deficiency at our center between 2000 and 2019; followed through 2025. Demographic, clinical, immunological, and follow-up data were retrospectively reviewed. Collected variables included gender, age, presenting symptoms, age at symptom onset and diagnosis, diagnostic delay, family history, infectious complications, physical examination findings, genetic characteristics, treatment modalities, clinical course, and outcomes. Laboratory evaluations included complete blood counts, serum immunoglobulin levels (IgG, IgA, IgM, and total IgE), assessment of specific antibody responses to vaccination (anti-hepatitis B surface antibody), and immunophenotypic analysis by flow cytometry. Immunoglobulin levels and lymphocyte subset results were evaluated according to age-matched reference ranges established by our laboratory (31–33). All patients were diagnosed according to the diagnostic criteria of the European Society for Immunodeficiencies (ESID) (34). The study was approved by the Ankara University Faculty of Medicine Human Research Ethics Committee (approval number: I01–98–26), and was conducted in accordance with the Declaration of Helsinki.

### Genetic analysis

Based on clinical findings and markedly reduced HLA-DR expression detected by flow cytometry, a targeted genetic analysis for MHC class II deficiency was performed. In the majority of patients (n=15), sequencing focused on the four genes known to regulate MHC class II expression (CIITA, RFXANK, RFX5, and RFXAP). However, in families with a previously identified pathogenic variant, genetic testing was restricted to targeted analysis of the known familial mutation. P1 in our cohort (corresponding to patient P2 in Matheux et al., 2002) was found to harbor a homozygous RFXAP (C→T) variant identified using a functional complementation assay with lentiviral vectors (35). Restoration of MHC class II expression following complementation confirmed the pathogenic role of the variant. P2, the sibling of P1, was subsequently diagnosed through family screening after P1 was identified by flow cytometry, and the diagnosis was genetically confirmed by targeted sequencing of RFXAP. In the remaining patients, coding regions of the *CIITA, RFX5, RFXAP*, and *RFXANK* genes were analyzed using next-generation sequencing panels. Whole-exome sequencing was not performed.

Genomic DNA was isolated using an automated magnetic separation system. Exome enrichment was performed using the Twist Comprehensive Human Exome kit, followed by sequencing on the MGI-T7 platform with a mean on-target depth of 80–100× and 150-bp paired-end reads. Bioinformatic analysis was performed using an in-house pipeline based on GATK best practices. Reads were aligned to the GRCh38 reference genome, and variants were called using GATK HaplotypeCaller. Copy number variations were assessed using GATK GermlineCNVCaller.

Identified variants were annotated and filtered based on allele frequency (minor allele frequency [MAF] <1%) in population databases and predicted pathogenicity using in silico tools (SIFT, PolyPhen-2, SpliceAI). Variant annotation was performed using Ensembl Variant Effect Predictor, and candidate variants were visually inspected using the Integrative Genomics Viewer (IGV). Variants were classified according to the American College of Medical Genetics and Genomics/Association for Molecular Pathology (ACMG/AMP) guidelines, applying established criteria (e.g., PVS1, PM2, PP3) in the context of variant type, population frequency, computational predictions, and clinical phenotype.

### Flow cytometric analysis

Flow cytometric analysis was performed on 2–3 mL EDTA-anticoagulated peripheral whole blood samples using a direct surface staining method and analyzed with Navios EX flow cytometer (Beckman Coulter, Miami, FL, USA). Peripheral blood lymphocyte subset analysis performed via CD45/SS gated cells included the following panel in patients diagnosed before 2020: Total T-cells (CD3^+^), CD4^+^ helper T cells (CD3^+^CD4^+^), CD8^+^ cytotoxic T cells (CD3^+^CD8^+^), naïve CD4^+^ T cells (CD3^+^CD4^+^CD45RA^+^), memory CD4^+^ T cells (CD3^+^CD4^+^CD45RO^+^), and γδ T cells (CD3^+^TCRγδ^+^). B cells were identified as CD19^+^ and/or CD20^+^ cells, and natural killer (NK) cells were defined as CD3^-^CD16^+^CD56^+^ cells. Surface expression of HLA-DR and HLA-ABC was also assessed primarily on CD45^+^ lymphocytes. Absent or markedly reduced values of HLA-DR expressions were further evaluated via staining the monocytes and/or activated T cells for confirmatory and accurate evaluation. Recent thymic emigrants (RTE) were defined as CD45+CD4^+^CD45RA^+^CD31^+^ T cells. In a small subset of patients (n=3) with long-term follow-up after HSCT (post-2020), a more detailed T-cell subset analysis was performed based on CD45RA and CCR7 expression on CD4 and CD8 positive T cells. CD4^+^ and CD8^+^ T cells were further classified into naïve (CD45RA^+^CCR7^+^), central memory (CD45RA^-^CCR7^+^), effector memory (CD45RA^-^CCR7^-^), and TEMRA (CD45RA^+^CCR7^-^) subsets. The B-cell subgroup analysis was also performed at variable time points in some patients (n=5) in whom post-HSCT follow-up data were available. B-cell subsets were defined as follows: naïve B cells (CD19^+^ CD27^-^IgD^+^), marginal zone B cells (CD19^+^ CD27^+^IgD^+^), switched memory B cells (CD19^+^ CD27^+^IgD^-^), and activated B cells (CD19^+^CD38^low^CD21^low^). Given the retrospective nature of the study, these analyses were not performed longitudinally or at standardized time points.

Antibody clones and suppliers are provided in **Supplementary Table 1**. Detailed definitions of T- and B-cell subsets are provided in **Supplementary Table 2**, and the results of detailed T-cell and B-cell subset analyses at last follow-up after HSCT are presented in **Supplementary Tables 3** and **4**, respectively.

### T-cell activation assay

T-cell activation responses were assessed following stimulation with phytohemagglutinin (PHA; Sigma-Aldrich, St. Louis, MO, USA). Peripheral blood mononuclear cells (PBMCs) were isolated from heparin-anticoagulated blood samples by density gradient centrifugation and cultured in RPMI-1640 medium supplemented with 10% fetal bovine serum, L-glutamine, and penicillin–streptomycin at a density of 1 × 10^5^ cells per tube.

PBMCs were stimulated with PHA at a final concentration of 10 µg/mL for 48 hours at 37 °C in a humidified atmosphere with 5% CO_2_. Following stimulation, T-cell activation was assessed by measuring CD25 and CD69 expression on T lymphocytes using flow cytometry. Results were interpreted according to age-matched reference ranges established in our laboratory.

### Treatment strategies and clinical outcomes

Data on therapeutic interventions and clinical outcomes were retrospectively collected. All patients received supportive management, including IgRT and antimicrobial prophylaxis. For patients who underwent HSCT, transplant-related characteristics were recorded, including donor type, age at transplantation, conditioning regimen, GvHD prophylaxis, and stem cell source, infused CD34^+^ cell dose, engraftment status, post-transplant complications, immune reconstitution, chimerism results, and survival outcomes.

According to European Society for Blood and Marrow Transplantation (EBMT) criteria, myeloablative conditioning (MAC) was defined as a busulfan-based regimen with a cumulative busulfan dose ≥14 mg/kg, in combination with cyclophosphamide (60 mg/kg/day for 2 days) or fludarabine (160 mg/m²). Reduced-toxicity conditioning (RTC) was defined as a treosulfan-based regimen (36–42 g/m², -7 to -5) in combination with fludarabine (150 mg/m², -7 to -3). Given the long study period, conditioning regimens were categorized according to EBMT/ESID definitions that were applicable at the time of transplantation (36).

### Engraftment and immune reconstitution definitions

Neutrophil engraftment was defined as the first of three consecutive days with an absolute neutrophil count (ANC) ≥500/mm³ Platelet engraftment was defined as the first of three consecutive days with a platelet count ≥20,000/mm³ in the absence of platelet transfusion for seven consecutive days (37). Immune reconstitution was evaluated based on recovery of HLA-DR expression on on CD45^+^ lymphocytes, with confirmatory assessment on monocytes when available (≥5%) and T-cell recovery, defined as CD3^+^ T-cell counts ≥50% and/or ≥1000/mm³ and CD4^+^ T-cell counts ≥25% and/or ≥500/mm³. B-cell reconstitution was defined as a CD19^+^ or CD20^+^ B-cell count ≥10% and/or ≥400/mm³, independent of immunoglobulin replacement therapy.

### Chimerism analysis

After HSCT, donor chimerism was assessed in peripheral blood using short tandem repeat (STR) analysis with the Plex Plus (100) PCR Assay Kit (Qiagen, Hilden, Germany). Myeloid and T-cell donor chimerism were evaluated at regular intervals according to the institutional follow-up protocol and the patients’ clinical availability. Full donor chimerism was defined as ≥ 95% donor-derived cells.

## Results

### Demographic and clinical features

We report 22 patients from 19 unrelated families (13 males and 9 females), all born to consanguineous parents. In three families, an affected sibling had previously been diagnosed with MHC class II deficiency at our center; two of these patients (P2 and P14) were identified through family screening before the onset of clinical symptoms, whereas one patient (P21) was diagnosed during the first infectious episode. Four families reported a history of sibling death due to severe infections without a prior diagnosis.

The median age at symptom onset was 9 months (range, 10 days–144 months), with a median age at diagnosis of 12 months (range, 10 days-50 months), yielding a median diagnostic delay of 7 months (range, 0–46 months). The wide range in age at symptom onset was largely driven by one patient (P2) with an atypical late-onset presentation identified through family screening. Excluding this patient, the median age at symptom onset was 2.8 months (range, 0.3–6.5 months) and the median age at diagnosis was 10 months (range, 0.3–48 months). Notably, diagnostic delay was markedly prolonged in one patient (P19) with a relatively mild clinical phenotype, who was diagnosed at 48 months despite symptom onset at 2 months. The mean follow-up duration was 64 months (range, seven days-300 months). Demographic and clinical characteristics of the patients are summarized in **Table 1**.

**Table 1:** Demographic, baseline clinical, genetic features and outcomes of patients with MHC class II deficiency.

Patient ID	Sex	Age at onset of symptoms (mo)	Age at diagnosis (mo)	Age at last follow-up (mo/yrs)	Presenting manifestations	Infectious agents at presentation	Chronic diarrhea	PICU at presentation	Causative genetic variant	HSCT (yes/no)	Current status
P1	M	2	8	10	Pneumonia, oral candidiasis, and aphthous ulcers, FTT	na	Yes	No	*RFXAP*	Yes	Died
P2	F	144 (12 yrs)	50 (4.2 yrs)	354/29.5	Asymptomatic (family screening)	No	No	No	*RFXAP:*	No	Alive
P3	F	2	6	12	Chronic diarrhea, oral candidiasis, pneumonia, FTT, hypotonia, mild dysmorphism	CMV	Yes	No	–	Yes	Died
P4	M	6	17	18	Severe pneumonia, encephalopathy, hyponatremia, and maculopapular rash	CMV	No	Yes	–	No	Died
P5	M	4	16	50/4.2	Recurrent pneumonia, chronic diarrhea, developmental delay, FTT	CMV, *C.parvum*	Yes	No	–	No	Died
P6	F	3	11	13	Severe pneumonia, respiratory distress, oral candidiasis, growth retardation, FTT	CMV, *P.jirevecii*	Yes	Yes	–	No	Died
P7	F	0,4	17	219/18.3	Chronic diarrhea, poor weight gain, FTT	CMV	Yes	No	–	Yes	Alive
P8	M	2	11	204/17	Recurrent pneumonia, developmental delay, optic atrophy, nystagmus, strabismus, FTT	Rotavirus	Yes	No	–	Yes	Alive
P9	F	0,3	9	194/16.2	Oral candidiasis, fever, recurrent viral pneumonia/bronchiolitis, BCG’itis	CMV	No	No	*RFXANK:*	Yes	Alive
P10	M	3	7	8	Severe pneumonia, FTT	CMV	No	No	*RFXANK*	No	Died
P11	M	3	4	11	Pneumonia, chronic diarrhea, oral candidiasis, pancytopenia, maculopapular rash, encephalopathy	CMV, RSV	Yes	No	*RFX5*	Yes	Died
P12	F	6	8	35	Oral candidiasis, growth retardation, pneumonia, FTT	HCoV-OC43, *S.pneumoniae* *H.influenzae*	Yes	No	*CIITA*	Yes	Died
P13	F	3	8	151/12.6	Severe pneumonia requiring tracheostomy, chronic diarrhea, oral candidiasis, FTT	*P. aeruginosa*	Yes	Yes	*RFX5*	Yes	Alive
P14	M	0,3	0,3	147/12.3	Family screening, developed pneumonia shortly after diagnosis	CMV, *P.Jirovecii*	No	No	*CIITA:*	Yes	Alive
P15	F	2	5	122/10.2	Purulent otitis, severe pneumonia, maculopapular rash, FTT	Rotavirus	No	No	*RFXANK*):	Yes	Alive
P16	M	4	5,5	5,5	Diarrhea, severe pneumonia, ARDS, FTT	Rhinovirus	Yes	Yes	–	No	Died
P17	M	6	7	8	Pneumonia, ARDS, stereotypic movements, altered consciousness, encephalopathy, hypotonia, mild facial dysmorhism, FTT	*P.Jirovecii*, Rhinovirus, HCoV-OC43	No	Yes	*CIITA*	No	Died
P18	M	1	6	23	Pneumonia, diarrhea, FTT, developmental delay, nystagmus	AdV, RSV, Rhinovirus	Yes	No	*RFXAP*):	Yes	Died
P19	M	2	48	192/16	Recurrent pneumonia, intermittant diarrhea, verruca plana, FTT	HPV	No	No	*RFXANK*	No	Alive
P20	F	2	7	7,5	Pneumonia, oral candidiasis, mild facial dysmorphism, epicanthus, hypotonia, FTT	na	Yes	Yes	*RFXAP*	No	Died
P21	M	6,5	7,5	8	Pneumonia, diarrhea, encephalopathy, FTT	AdV, RSV	Yes	No	*RFX5*	No	Died
P22	M	2	3,5	4	Pneumonia, ARDS, septic shock, myocarditis	CMV	No	Yes	*RFXANK:*	No	Died

PICU, Pediatric intensive care unit; HSCT, hematopoietic stem cell transplantation; FTT, Failure to thrive; HCoV-OC43, Human coronavirus OC43; AdV, Adenovirus; ARDS, acute respiratory distress syndrome; na, not available.

Pneumonia (n=19, 86%), failure to thrive (n=16, 72%), chronic diarrhea (n=13, 59%) were the most common initial manifestations. Hepatomegaly was detected in 13 patients (59%), with elevated liver enzymes in 10 (45%). Gastrointestinal manifestations were frequent and included growth failure and chronic diarrhea. Hepatobiliary complications were rare; only one patient had documented *Cryptosporidium parvum* infection and died due to hepatic encephalopathy, and no patients developed sclerosing cholangitis. Less frequent findings included mild facial dysmorphism, ocular abnormalities, and neurodevelopmental manifestations, including developmental delay at diagnosis and neurological features such as hypotonia and ataxia. Encephalopathy during severe systemic infection was observed in four patients. Autoimmune features were rare (4,5%), limited to autoimmune thyroiditis and adrenal insufficiency. Cutaneous manifestations were documented in 9 patients (41%), predominantly maculopapular eruptions, most often occurring during broad-spectrum antibiotic use. Fourteen patients required pediatric intensive care unit (PICU) admission due to severe infections (seven at initial presentation and seven during follow-up). Among patients requiring PICU admission at initial presentation, mortality was high (6/7), and only one patient survived following HSCT.

### Genetic diagnosis

Genetic analysis was performed in 15 patients all of whom were analyzed for variants in the four known causative genes (RFXANK, RFXAP, RFX5, and CIITA). Homozygous pathogenic variants were identified in one of these genes in each patient, distributed as follows: *RFXANK* (n = 5), *RFXAP* (n = 4), *RFX5* (n = 3), and *CIITA* (n = 3).

In patient P1 (corresponding to P2 in Mathieux et al., 2002), a previously reported *RFXAP* (C>T) mutation was confirmed using a functional complementation assay. The characteristics of the identified mutations, including gene, cDNA and protein changes, variant type, zygosity, method, ACMG classification, and previous reports, are summarized in **Table 2**. Four novel pathogenic variants were identified in six patients A novel homozygous frameshift variant in *CIITA* (c.277del; p.Glu93ArgfsTer22), which was predicted to result in premature truncation of the *CIITA* protein, was identified in two siblings P12 and P14. In patient P15, a previously unreported homozygous missense variant in *RFXANK* (c.395C>T; p.Ser132Phe) was identified. In addition, a novel homozygous nonsense variant in *RFXAP* (c.757C>T; p.Gln253Ter), predicted to result in a premature stop codon, was identified in patient P18. A homozygous deletion involving exon 1 of the *RFXAP* gene was detected in patient P20; this pathogenic variant was consistent with and supported the clinical diagnosis. No evidence of a founder effect was observed. Genetic analysis could not be performed in the remaining seven patients due to insufficient biological material or early death before testing could be completed.

**Table 2:** Detailed genetic findings in patients with MHC class II deficiency.

Patient*	Gene	cDNA	Protein	Variant type	Zygosity	Method	ACMG class	ACMG criteria	rsID	Previous report
P1	RFXAP	c.751C>T	p.Gln251*	Non sense (stop codon)	Homozygous	Functional	Pathogenic	PVS1, PM2, PS3	NA	Ref. 35 (novel)
P2	RFXAP	c.751C>T	p.Gln251*	Non sense (stop codon)	Homozygous	Targeted SS	Pathogenic	PVS1, PM2, PS3	NA	Previously reported
P9	RFXANK	c.634C>T	p.Arg212*	Non sense (stop codon)	Homozygous	Targeted NGS panel	Pathogenic	PVS1, PM2	rs747402973	Previously reported
P10	RFXANK	c.304G>T	p.Glu102*	Non sense (stop codon)	Homozygous	Targeted NGS panel	Pathogenic	PVS1, PM2	rs760362888	Previously reported
P11	RFX5	c.1198C>T	p.Arg400*	Non sense (stop codon)	Homozygous	Targeted NGS panel	Pathogenic	PVS1, PM2	rs766158684	Reported (no previous disease association)
P12	CIITA	c.277del	p.Glu93Argfs*22	Frameshift+ stop codon	Homozygous	Targeted NGS panel	Pathogenic	PVS1, PM2	NA	Novel (P12 and P14 are sibling)
P13	RFX5	c.616G>C	p.Ala206Pro	Missense	Homozygous	Targeted NGS panel	Likely pathogenic	PVS1, PM2, PP3	rs760362888	Previously reported
P14	CIITA	c.277del	p.Glu93Argfs*22	Frameshift+ stop codon	Homozygous	Targeted SS	Pathogenic	PVS1, PM2,	NA	Novel (P12 and P14 are sibling)
P15	RFXANK	c.395C>T	p.Ser132Phe	Missense	Homozygous	Targeted NGS panel	VUS	PM2, PP3	NA	Novel
P17	CIITA	c.922C>T	p.Arg308*	Non sense (stop codon)	Homozygous	Targeted NGS panel	Pathogenic	PVS1, PM2	rs567218474	Previously reported
P18	RFXAP	c.757C>T	p.Gln253*	Non sense (stop codon)	Homozygous	Targeted NGS panel	Pathogenic	PVS1, PM2	NA	Novel
P19	RFXANK	c.752_767del	p.Gln251Profs?*	Frameshift/deletion	Homozygous	Targeted NGS panel	Pathogenic	PVS1, PM2, PP4	NA	Novel
P20	RFXAP	Exon 1 deletion	Predicted loss of function	Exonic deletion	Homozygous	Targeted NGS panel	Pathogenic	PVS1, PM2, PP4	NA	Novel
P21	RFX5	c.1198C>T	p.Arg400*	Non sense (stop codon)	Homozygous	Targeted SS	Pathogenic	PVS1, PM2	rs766158684	Reported (no previous disease association)
P22	RFXANK	c.634C>T	p.Arg212*	Non sense (stop codon)	Homozygous	Targeted NGS panel	Pathogenic	PVS1, PM2	rs747402973	Previously reported

*: Indicates a stop codon leading to premature termination of the protein.

### Infections

Recurrent infections were the most common features. Viral pathogens included CMV (n=11), rhinovirus (n=3), RSV (n=3), adenovirus (n=2), rotavirus (n=2), human coronavirus OC43 (n=2), SARS-CoV-2 (n=1), and enterovirus (n=1). Bacterial and opportunistic infections were also frequent, including *Pseudomonas aeruginosa* (n=3), *Streptococcus pneumoniae* (n=1), oral candidiasis (n=8), *Candida* urinary tract infection (n=1), *Candida* esophagitis (n=1), *Pneumocystis jirovecii* pneumonia (PJP) (n=3), and *Cryptosporidium parvum* gastroenteritis (n=1). One patient (P19) developed extensive HPV-associated warts. BCG vaccination had been administered to 17 patients before the diagnosis, with local BCG-itis observed in three cases (one pre- and two post-transplant). All tested patients (18/18) were HIV-negative. Severe viral infections were more frequently observed among patients with fatal outcomes, both in transplanted and non-transplanted groups.

### Immunological characteristics

Immunological characteristics are summarized in **Table 3**. Flow cytometry demonstrated absent or markedly reduced HLA-DR expression on CD45+ lymphocytes and monocytes, with low-level residual expression detectable on activated T cells only in a small subset of patients (P2 and P19) who presented with an atypical and milder clinical phenotype. At diagnosis, 14/22 patients (63.6%) had normal lymphocyte counts, while 8/22 (36%) were lymphopenic. CD4^+^ T-cell lymphopenia was detected in 18/22 patients (82%), with reduced naïve CD4^+^CD45RA^+^ subsets in 16/22 (73%). RTE counts were mildly to moderately below age-matched reference ranges in all 12 tested patients, indicating impaired thymic output; concordantly, chest imaging revealed an absent or small thymic shadow in 6/12 patients.

**Table 3:** Immunological characteristics of patients with MHC class II deficiency.

Pt. No	WBC (mm^3^)	TLS (mm^3^)	CD3^+^ T cells (%/mm^3^)	CD3^+^CD4^+^ T cells (%/mm^3^)	CD8^+^ T cells (%/mm^3^)	CD19^+^ B cells (%/mm^3^)	CD3 CD16^+^CD56^+^ NK cells (%/mm^3^)	RTE (%)	HLA-DR exp. (%) (CD45+ lymphocyte/monocytes / activated T cells)	HLA-ABC exp. (%)	Lymp. activation response to PHA	IgG (mg/dl)	IgA (mg/dl)	IgM (mg/dl)	Total IgE kU/l
P1	3500	2000	58 (51–79) 1160 (2400–6100)	28 (31–54) 560 (1400–5200)	38 (10–31) 760 (600–3000)	28 (14–44) 560 (500–3600)	15 (5–23) 300 (200–1800)	nd	0/0/0	nd	Normal	125 (304–1231)	8 (7–123)	19 (32–203)	nd
P2	6200	2300	85 (55–79) 1955 (1900–3600)	12 (26–49) 276 (600–2000)	65 (9–36) 1495 (300–1300)	5 (11–31) 115 (300–1200)	5 (5–28) 115 (200–1200)	13 (31–81)	0/0/2.9–0 **	60	Normal	443 (745–1804)	78 (57–282)	59 (78–261)	2,9
P3	12800	7900	60 (51–79) 4740 (2400–6100)	10 (31–54) 790 (1400–5200)	44 (10–31) 3480 (600–3000)	33 (14–44) 2600 (500–3600)	14 (5–23) 1100 (200–1800)	nd	0/0/0	83	Normal	155 (304–1231)	23 (7–123)	83 (32–203)	14
P4	4300	1550	64 (51–77) 992 (1300–6500)	11 (29–55) 170 (700–4500)	49 (15–33) 760 (400–3200)	27 (17–41)/ 418 (500–3600)	3 (4–15) 47 (200–1300)	nd	0/0/0	nd	nd	412 (605–1430)	43 (30–107)	71 (66–228)	<5
P5	9600	5300	76 (51–77) 4028 (1300–6500)	15 (29–55) 795 (700–4500)	49 (15–33) 2600 (400–3200)	20 (17–41) 1060 (500–3600)	3 (4–15) 159 (200–1300)	nd	0/0/0	87	Low	507 (605–1430)	22 (30–107)	17 (66–228)	nd
P6	9900	2400	66 (51–79) 1584 (2400–6100)	22 (31–54) 528 (1400–5200)	43 (10–31) 1032 (600–3000)	21 (14–44) 504 (500–3600)	4 (5–23) 96 (200–1800)	nd	0/0/0	98	nd	687* (463–1006)	22 (17–69)	63 (46–159)	nd
P7	14600	7900	73 (51–77) 5767 (1300–6500)	10 (29–55) 790 (700–4500)	68 (15–33) 5372 (400–3200)	23 (17–41) 1817 (500–3600)	2,8 (4–15) 221 (200–1300)	nd	0/0/0	97	Low	177 (605–1430)	22 (30–107)	32 (66–228)	20
P8	4700	3900	71 (51–79) 2770 (2400–6100)	24 (31–54) 936 (1400–5200)	24 (10–31) 936 (600–3000)	20 (14–44) 780 (500–3600)	1 (5–23) 39 (200–1800)	nd	0/0/0	86	Normal	1410* (463–1006)	22 (17–69)	17 (46–159)	4,5
P9	9300	6900	61 (51–79) 4209 (2400–6100)	13 (31–54) 897 (1400–5200)	48 (10–31) 3312 (600–3000)	35 (14–44) 2415 (500–3600)	1 (5–23) 69 (200–1800)	nd	0/0/0	82	Normal	185 (463–1006)	<24 (17–69)	63 (46–159)	14
P10	19400	13800	77 (51–79) 10600 (2400–6100)	12 (31–54) 1656 (1400–5200)	51 (10–31) 7038 (600–3000)	16 (14–44) 2208 (500–3600)	3 (5–23) 414 (200–1800)	nd	0/0/0	nd	Normal	1240* (304–1231)	59 (7–123)	121 (32–203)	nd
P11	3300	1400	79 (51–79) 1100 (2400–6100)	15 (31–54) 210 (1400–5200)	62 (10–31) 868 (600–3000)	8 (14–44) 112 (500–3600)	10 (5–23) 140 (200–1800)	nd	0/0/0	nd	Normal	154 (304–1231)	16 (7–123)	17 (32–203)	nd
P12	7800	4100	61 (51–79) 2500 (2400–6100)	15 (31–54) 615 (1400–5200)	40 (10–31) 1640 (600–3000)	22 (14–44) 902 (500–3600)	14 (5–23) 574 (200–1800)	41 (63–81)	0/0/0	100	Normal	317 (304–1231)	<5 (7–123)	75 (32–203)	0,6
P13	15800	6300	62 (51–79) 2852 (2400–6100)	17 (31–54) 782 (1400–5200)	46 (10–31) 2116 (600–3000)	33 (14–44) 1518 (500–3600)	2,6 (5–23) 120 (200–1800)	13 (57–82)	0/0/0	100	Normal	874* (304–1231)	12 (7–123)	<16 (32–203)	<2
P14	9600	4200	80 (51–79) 3360 (2400–6100)	15 (31–54) 630 (1400–5200)	65 (10–31) 2730 (600–3000)	5 (14–44) 210 (500–3600)	14 (5–23) 588 (200–1800)	29 (59–81)	0/0/0	nd	Normal	488 (633–1466)	<5	<16 (22–87)	<2
P15	7500	1800	56 (51–79) 1008 (2400–6100)	32 (31–54) 576 (1400–5200)	19 (10–31) 342 (600–3000)	41 (14–44) 738 (500–3600)	3 (5–23) 168 (200–1800)	43 (63–81)	0/0/0	78	Normal	137 (304–1231)	<25 (7–123)	19 (32–203)	<2
P16	7230	4680	35 (51–79) 1638 (2400–6100)	7 (31–54) 328 (1400–5200)	25 (10–31) 1170 (600–3000)	64 (14–44) 889 (500–3600)	3 (5–23) 140 (200–1800)	18 (63–81)	0/0/0	98	Low	<33 (304–1231)	<6 (7–123)	15 (32–203)	<5
P17	24180	6620	24 (51–79) 1588 (2400–6100)	5 (31–54) 331 (1400–5200)	18 (10–31) 1192 (600–3000)	75 (14–44) 4965 (500–3600)	0.2 (5–23) 13 (200–1800)	25 (63–81)	0/0/0	100	Normal	<33 (304–1231)	<6 (7–123)	104 (32–203)	<5
P18	6080	2170	89 (51–79) 1931 (2400–6100)	16 (31–54) 347 (1400–5200)	70 (10–31) 1519 (600–3000)	4 (14–44) 87 (500–3600)	5 (5–23) 108 (200–1800)	55 (63–81)	0/0/0	71	Normal	1120* (304–1231)	<6 (7–123)	<6 (32–203)	<2
P19	16500	5830	74 (55–79) 4310 (1900–3600)	20 (26–49) 1166 (600–2000)	48 (9–36) 2798 (300–1300)	10 (11–31) 538 (300–1200)	7 (5–28) 408 (200–1200)	21 (53–69)	1/1/2,4-4**	88	Normal	1200* (640–2010)	21 (44–244)	185 (52–297)	14
P20	3710	1630	36 (51–79) 587 (2400–6100)	12 (31–54) 196 (1400–5200)	23 (10–31) 378 (600–3000)	45 (14–44) 733 (500–3600)	18 (5–23) 293 (200–1800)	50 (63–81)	0	80	Low	122 (304–1231)	<6 (7–123)	17 (32–203)	<5
P21	5760	1728	58 (51–79) 1000 (2400–6100)	9 (31–54) 160 (1400–5200)	43 (10–31) 765 (600–3000)	21 (14–44) 373 (500–3600)	15 (5–23) 259 (200–1800)	16 (59–81)	0	88	Normal	<33 (304–1231)	<6 (7–123)	61 (32–203)	nd
P22	15420	5410	42 (51–79) 1220 (2400–6100)	6 (31–54) 175 (1400–5200)	35 (10–31) 960 (600–3000)	46 (14–44) 1388 (500–3600)	5 (5–23) 146 (200–1800)	35 (59–81)	0	100	Low	226 (304–1231)	<6 (7–123)	40 (32–203)	<17

*: IgG levels were measured while on IgRT, **: Values represent HLA-DR expression on activated T cells measured at two different time points during follow-up.

CD8^+^ T-cell percentages were elevated in 17/22 patients (77%), although absolute counts were normal in most (11/17). The CD4/CD8 ratio was inverted in 20/22 patients (91%). Increased TCR γδ T-cell frequencies were observed in four patients, including patients with milder clinical phenotypes. T-cell activation assessed by phytohemagglutinin (PHA) stimulation was generally preserved, with reduced responses observed in some critically ill individuals. NK cells were reduced in 13/22 (59%).

B-cell counts were normal or increased in 16/22 patients (73%). Hypogammaglobulinemia was common: IgG and IgA were reduced in 15/22 patients (68% each), and IgM in 12/22 (55%). Vaccine-specific antibody responses were largely absent, and isohemagglutinin titers were negative in 11/12 tested patients. HLA-ABC expression was reduced in 10/17 (59%). Eosinophilia at diagnosis was observed in seven patients (range: 600 to 5.500/mm³).

### HSCT outcomes

Transplantation characteristics and outcomes are summarized in **Table 4**. Ten patients (45%) underwent HSCT at our center. Median age at transplant was 10 months (range, 7–29 months), with a median interval from diagnosis to transplantation of 2 months (range, 2–18 months). In one patient (P8), HSCT was postponed due to active tuberculosis in the donor and was performed after completion of anti-tuberculosis therapy. One additional patient (P11) underwent HSCT at another center and died early in the post-transplant period; therefore, this patient was excluded from subsequent transplant outcome analyses.

**Table 4:** HSCT characteristics and outcomes.

Patient No	Age at HSCT (mo)	Donor type	Stem cell source	Conditioning regimen	GvHD prophylaxis	Infused CD34^+^ cell dose (×10^6^/kg)	Days to HLA-DR recovery (CD45+ lymphocyte/monocytes)	HLA-DR expression at engraftment (%)	Days to neutrophil engraftment	Days to platelet engraftment	Acute GvHD	Chronic GvHD	Other HSCT-related complications	Outcome at last follow-up
P1	10	Haplo.	PBSC	Flu+Mel+ATG+Bu	CsA+MMF	24,5	13/na	23	13	–	No	–	VOD, sepsis	Died on day +17 post-HSCT due to VOD and sepsis
P3	9	MRD	BM	Bu 4 mg/kg × 4 days Cyclo 60mg/kg × 2 days	CsA	5	13/na	57	12	17	Grade 2 skin and intestinal	–	CMV reactivation, sepsis, intracranial hemorrhage	Died 3 months post-HSCT (intestinal GvHD, sepsis, DIC, intracranial hemorrhage).
P7	19	MSD	BM	Bu 4 mg/kg × 4 days Cyclo 60mg/kg × 2 days	CsA	13	16/na	34	14	16	Grade 2 skin	No	CMV reactivation, septic artritis, hearing loss	Alive at last follow-up (+16 years post-HSCT); currently 17 years old; using a hearing aid
P8	29	MSD	PBSC	Bu 4 mg/kg × 4 days Cyclo 60mg/kg × 2 days	CsA	11,5	14/na	27	13	15	No	No	Persistent neurodevelopmental impairment, severe growth failure, strabismus	Alive at last follow-up (+15 years post-HSCT); currently 17 years old; with speech impairment and toe walking.
P9	11	MSD	BM	Bu 4 mg/kg × 4 days Cyclo 60mg/kg × 2 days	CsA + MTX	13	14/na	67	12	17	Grade 2 skin	No	Engraftment syndrome, CMV reactivation, CMV retinitis with sequelae, strabismus	Alive at last follow-up (+15 years post-HSCT); currently 16 years old; with left-eye visual impairment and strabismus.
P12	11	MRD	BM	Bu 4 mg/kg × 4 days Flu 40mg/m^2^/g× 4 days	CsA + MTX	5,1	21/na	48	17	38	No	–	RSV pneumonia, secondary graft failure, AIHA, CMV infection, hypertensive encephalopathy	Died at 24 months post-HSCT due to secondary graft failure and sepsis.
P13	13	MRD	BM	Treo 42 g/m^2^ (-7-5days) Flu 150mg/m^2^ (-7–3 days)	CsA + Mtx	7	10/30,3	74	10	15	Grade 2 skin	Yes	BCG-itis, CMV, vitiligo, bronchiolitis obliterans, bronchiectasis, hearing loss	Alive; bronchiolitis obliterans, bronchiectasis; learning difficulties; hearing aid use.
P14	7,5	MSD	BM	Treo 36 g/m^2^ (-7–5 days) Flu 150mg/m^2^ (-7–3days)	CsA	8,3	30/95	95	14	14	No	No	CMV reactivation	Alive, and well
P15	7	MRD	BM	Treo 36 g/m^2^ (-7–5 days) Flu 150mg/m^2^ (-7–3 days)	CsA + MTX	9	21/97	97	15	18	No	No	BCG-itis, CMV reactivation	Alive, and well
P18	8	MRD	PBSC	Treo 36 g/m^2^ (-7–5 days) Flu 150mg/m^2^ (–7–3 days)	CsA + MMF	10	12/24	22	12	22	Grade 3 skin, intestinal and liver	Yes	Acute and chronic GvHD (skin, liver, intestinal), secondary graft failure; PJP; sepsis	Died at +14 months post–HSCT due to chronic GvHD, PJP, and sepsis

PBSC, Peripheral blood stem cell; GvHD, Graft versus host disease; HSCT, Hematopoietic stem–cell transplantation; Flu, Fludarabine; Mel, Melfalan; ATG, Anti Thymoglobulin; Bu, Busulfan; CsA, Cyclosporin A; MMF, Mycophenolate mofetil; MRD, Matched related donor; BM, Bone marrow; Cyclo, cyclophosphamide; VOD, Veno–occlusive disease; DIC, disseminated intravascular coagulation; MSD, Matched sibling donor; MTX, Methotrexate; Treo, Treosulfan; PJP, *Pneumocystis jiroveci* pneumonia; na, not avaliable.

Donors were matched related donors in nine patients and a haploidentical donor in one case. Stem cell sources were bone marrow (n=7) and peripheral blood (n=3). Six patients (60%) received MAC and four (40%) received RTC. GvHD prophylaxis included cyclosporine ± methotrexate or mycophenolate mofetil (MMF). Anti-thymocyte globulin (ATG) was administered as serotherapy in the haploidentical transplant setting. The median CD34^+^ stem cell dose infused was 9.2 × 10^6^ cells/kg (range: 5–24.5 × 10^6^ cells/kg).

Neutrophil engraftment, platelet engrafment, and reconstitution of HLA-DR expression on CD45+ lymphocytes and, when available monocytes wereachieved in all patients, with a median time of 13 days (range, 10–17) 19 days (range, 14-38) and 16 days (range, 10–30), respectively.

Among 10, two patients died within the first 100 days following HSCT due to early transplant-related complications: veno-occlusive disease (VOD) with sepsis (P1) and sepsis complicated by severe intestinal GvHD (P3). Two additional patients died later due to secondary graft failure and severe infection (P12 and P18). P12 experienced graft loss followed by episodes of autoimmune hemolytic anemia and CMV reactivation, and died of sepsis at +24 months post-transplant. P18 developed chronic skin and intestinal GvHD, experienced secondary graft failure, and died of PJP and sepsis at +15 months. Acute grade II GvHD occurred in five patients (50%), and chronic GvHD in two (25%).

Early post-transplant BCG-itis was observed in two patients (P13 and P15). No severe invasive infections were observed during long-term follow-up. CMV viremia occurred in 7/10 patients, including five reactivations and two primary infections (P12, P13). One patient (P9), who had documented CMV infection prior to HSCT, developed CMV retinitis during post-transplant follow-up, resulting in permanent visual impairment and secondary strabismus.

Among survivors, chronic lung disease was documented in two patients, including bronchiolitis obliterans in one (P13) and bronchiectasis in two (P13 and P15). Both patients had a history of severe pneumonia prior to HSCT. P13 underwent transplantation after a critical pulmonary infection requiring prolonged mechanical ventilation and tracheostomy. Although the tracheostomy was successfully decannulated following engraftment, chronic pulmonary sequelae persisted during follow-up.

Neurological and neurodevelopmental sequelae were observed in a subset of patients. Two patients (P8 and P18) had pre-existing neurodevelopmental delay and nystagmus at diagnosis and subsequently developed post-transplant strabismus. One of these patients (P8) had persistent neurodevelopmental impairment, characterized by absent speech and toe walking. Post-transplant sensorineural hearing loss was observed in two patients (P7 and P13), both of whom required hearing devices; P13 also demonstrated learning difficulties and poor school performance.

The overall survival after HSCT was 60% (6/10). The 15-year OS was 50% (3/6) among MAC recipients, whereas the 10-year OS was 75% (3/4) in the RTC group. MAC was administered before 2014, while RTC was used from 2015 onwards. Patients in the MAC group underwent HSCT at a higher mean age than those receiving RTC (14.0 vs. 8.8 months). Severe transplant-related complications and long-term sequelae, including hearing and visual impairment, were more frequently observed in MAC recipients. Formal statistical comparisons were not performed due to the small sample size.

### Long-term outcomes and immune reconstitution

**Figure 1:**
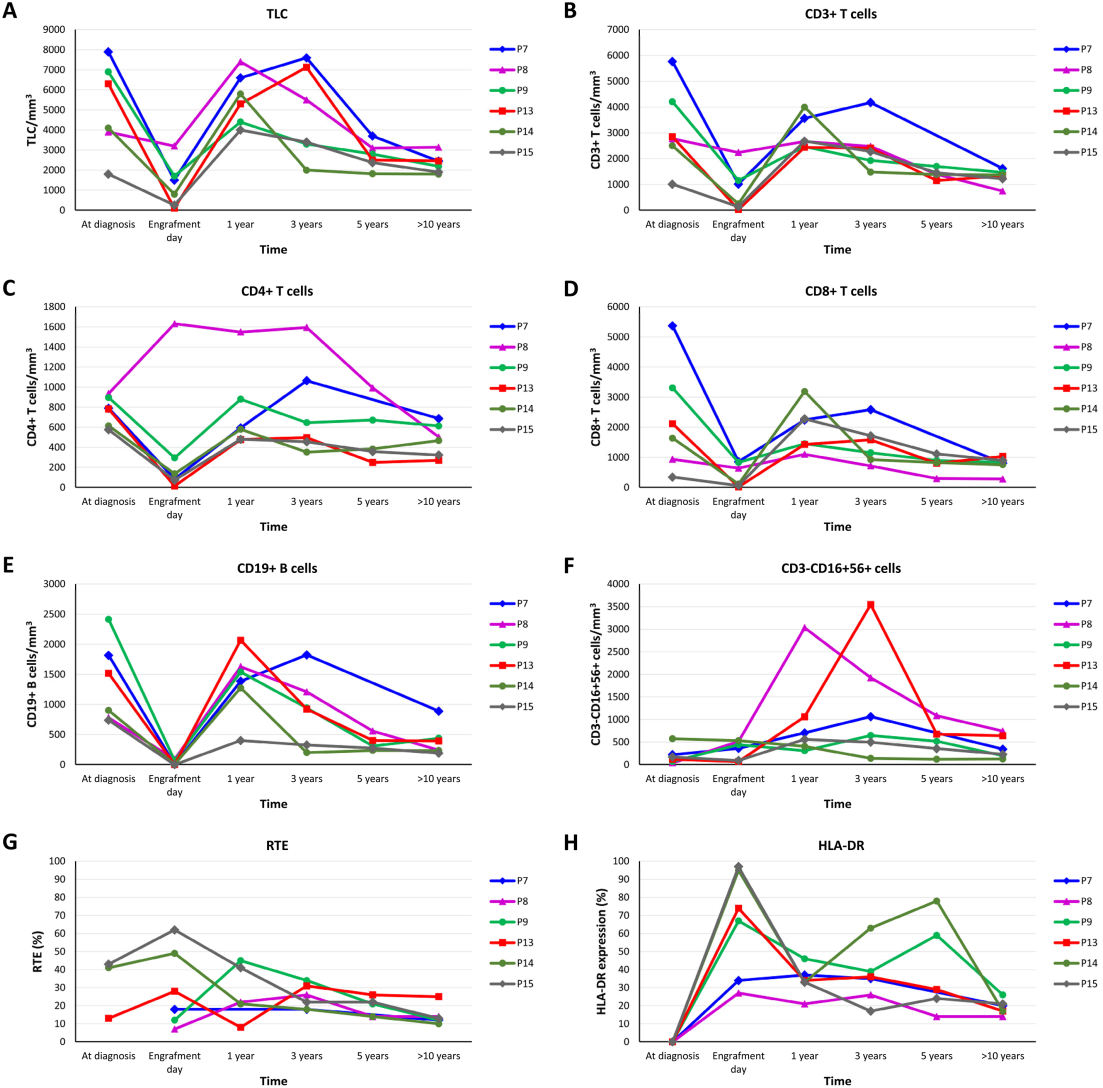
Long–term immune reconstitution following HSCT in MHC class II deficiency. Lymphocyte subsets at diagnosis and long-term post-HSCT follow-up. **(A)** Total lymphocyte count (TLC), **(B)** CD3^+^ T cells, **(C)** CD4^+^ T cells, **(D)** CD8^+^ T cells, **(E)** CD19^+^ B cells, **(F)** CD3^-^CD16^+^CD56^+^ NK cells, **(G)** recent thymic emigrants (RTE, and **(H)** HLA–DR expression (percentage) was measured on CD45^+^ lymphocytes at diagnosis, at engraftment, 1 year, 3 years, 5 years, and >10 years post–HSCTExcept for NK cells, most parameters showed a peak early after transplant followed by a slight decline over time.

Immune reconstitution data for long-term survivors, defined as patients surviving ≥10 years after HSCT (n=6), are summarized in **Table 5**. Total lymphocyte counts and CD3^+^, CD4^+^, CD8^+^, and CD19^+^ cell counts peaked at 1 year post-HSCT, followed by a slight decrease over long-term follow-up In contrast, NK-cell counts remained relatively stable over time (**Figure 1**). Total CD3^+^ T-cell counts were within the normal range at 1 year post-HSCT and remained normal at last follow-up in all except P8. At 1 year post-HSCT, the median CD4^+^ T-cell proportion was 11% (range, 9–21), with a median absolute count of 587/mm³ (range, 477–1150). At last follow-up, the median CD4^+^ T-cell proportion and absolute count were 27% (range, 11–28) and 485/mm³ (range, 270–686), respectively. CD4^+^ T-cell recovery remained heterogeneous, with consistently higher CD4^+^ T-cell counts observed in MAC recipients compared with RTC recipients at both 1 year post-HSCT and at last follow-up.

**Table 5:** Longitudinal immunereconstitution features of MHC class II deficiency patients.

Patient no	P7	P8	P9	P13	P14	P15	Age references
Age at HSCT (mo)	19	29	11	11	7,5	7	
Current status	Alive	Alive	Alive	Alive	Alive	Alive	
Last follow-up time (years post-HSCT)	+17	+ 15	+15	+11	+11	+10	
IgG (mg/dl) 1^st^ Year	912	1190	647	1020	1130	940	(605–1430)
Last follow-up	1060	909	943	971	1010	1080	(913–1884)
IgA (mg/dl) 1^st^ Year	24	95	13	59	37	36	(30–107)
Last follow-up	242 (88–322)	61	100	<23	256	260	(139–378)
IgM (mg/dl) 1^st^ Year	87 (66–228)	48 (52–297)	46 (66–228)	116 (66–228)	79 (66–228)	50	(66–228)
Last follow-up	124 (88–322)	61 (88–322)	50 (88–322)	82 (47–484)	57 (47–484)	101 (47–484)	
IgRT need after 1 year	No	No	No	No	No	No	
Vaccine responses after revaccination	Anti HBs IgG (+)	Anti HBs IgG (+)	Anti-HBs IgG (+)	Anti-HBs IgG (+)	Anti-HBs IgG (+)	Anti-HBs IgG (+)	
HLA-DR (%) (CD45+lymphocyte/monocyte) 1^st^ Year	37/na	21/na	46/na	34/na	33/na	33/na	
Last follow-up	20/76	14/91	26/58	17/38	17/48	21/90	
Total lymphocyte count (cells/mm³) 1^st^ Year	6600 (1500–5200)	7400 (1500–5200)	4400 (2200–8100)	5300 (2200–8100)	5800 (2200–8100)	4000 (2200–8100)	
Last follow-up	2450	3140	2190	2460	1810	1900	(1700–5700)
CD3^+^ T cells (%) (cells/mm³) 1^st^ Year	54 (55–79) 3564 (1900–3600)	36 (55–79) 2664 (1900–3600)	56 (51–77) 2464 (1300–6500)	46 (51–77) 2438 (1300–6500)	69 (51–77) 4000 (1300–6500)	67 (51–77) 2680 (1300–6500)	
Last follow–up	66 1617	40 744	67 1467	54 1328	76 1368	64 1216	(58–82) (1100–4100)
CD3^+^CD4^+^ T cells (%) (cells/mm³) 1^st^ Year	9 (26–49) 594 (600–2000)	21 (26–49) 1550 (600–2000)	20 (29–55) 880 (700–4500)	9 (29–55) 477 (700–4500)	10 580 (700–4500)	12 480 (700–4500)	
Last follow-up	28 686	27 502	28 613	11 270	26 468	17 323	(26–48) (600–2400)
CD3^+^CD8^+^ Tt cells (%) (cells/mm³) 1^st^ year	34 (9–35) 2244 (300–1300)	15 (9–35) 1110 (300–1300)	33 (15–33) 1452 (300–1300)	27 (15–33) 1431 (400–3200)	55 (15–33) 3190 (400–3200)	57 (15–33) 2280 (400–3200)	
Last follow-up	33 808	15 279	38 832	42 1033	42 756	47 893	(16–32) (400–1500)
CD3^-^CD16^+^CD56^+^ NK cells (%) (cells/mm³) 1^st^ Year	19 (5–26) 703 (200–1200)	41 (5–28) 3034 (200–1200)	7 (4–15) 308 (200–1300)	20 (4–15) 1060 (200–1300)	7 (4–15) 406 (200–1300)	14 (4–15) 560 (200–1300)	
Last follow-up	14 343	40 744	9 197	26 640	7 126	12 228	(8–30) (200–1000)
CD19+ B cells (%) (cells/mm³) 1^st^ Year	21 (11–31) 1386 (300–1200)	22 (11–31) 1628 (300–1200)	35 (17–41) 1540 (500–3600)	39 (17–41) 2067 (500–3600)	22 (17–41) 1276 (500–3600)	10 (17–41) 400 (500–3600)	
Last follow-up	9 888	13 241	20 438	16 393	13 234	10 190	(6–19) (200–1400)
CD3^+^CD45RA^+^CD31^+^ (RTE) (%) 1^st^ Year	nd	22	45	8	21	41	
Last follow-up	12	14	12	25	10	13	
Chimerism (% Myeloid cell/% T cell) 1^st^ Year	100/100	99/99	100/100	69/50	97/97	46/90	
3^rd^ year		98/98	85/100	60/62	nd/nd	9/83	
5^th^ year	–	98/98	90/98	49/59	97/97	11/87	
≥10^th^ year	nd/84	nd/80	nd/90	53/49	96/96	30/81	

na, not available; nd, not determined.

At 1 year post-HSCT, the median CD8^+^ T-cell proportion was 34% (range, 15–57) and the median absolute count was 1848/mm³ (range, 1110–3190); at last follow-up, the corresponding values were 40% (range, 10–42) and 820/mm³ (range, 314–1260), respectively.

HLA-DR expression showed recovery, with a median of 33% (range, 21–46) at 1 year post-HSCT and and fluctuated during long-term follow-up (range, 10–97). Naïve T-cell reconstitution remained limited, as reflected by persistently reduced RTE. The median proportion of RTE was 22% (range, 5–45) at 1 year post-HSCT and 19% (range, 14–35) at last follow-up, remaining below age-matched reference ranges in all evaluated survivors. HLA-ABC expression was within the normal range in all surviving patients at last follow-up. Definitions of T-cell subsets used for immunophenotyping are provided in **Supplementary Table 2**. Detailed T-cell subset analysis, including naïve, central memory, effector memory, and TEMRA populations, was available in three patients and is summarized in **Supplementary Table 3**.

In contrast, CD19^+^ B-cell reconstitution was adequate in all long-term survivors, allowing discontinuation of IgRT. Protective vaccine-specific antibody responses were achieved in most survivors. Post-HSCT B-cell subset analysis was available in five patients at variable time points. Mild abnormalities were observed in specific B-cell compartments: switched memory B cells were reduced relative to age-matched reference ranges in one patient (P13), marginal zone B cells were borderline low in two patients (P13 and P15) and decreased in one patient (P7), and naïve B cells were reduced in two patients (P7 and P9). However, none of the patients experienced recurrent or severe infections or required re-initiation of immunoglobulin replacement therapy following HSCT.

Chimerism analysis demonstrated full donor chimerism within the 1 year in all MAC recipients, whereas mixed chimerism was observed in two of three RTC recipients. At last follow-up, T-cell chimerism was mixed in both conditioning groups. Myeloid chimerism data were available for three patients at last follow-up and could not be assessed in the remaining cases.

### Outcome in non-transplanted patients

Among 11 patients who did not undergo HSCT, nine died, resulting in an HSCT-free survival of 18%. All deceased patients exhibited a SCID-like clinical phenotype and died before one year of age, consistent with the natural history of classical SCID without transplantation. Most presented with severe infections, including CMV (n=5), rhinovirus (n=2), adenovirus/RSV co-infection (n=1), as well as severe respiratory failure (ARDS) with encephalopathy (**Table 1**). In contrast, the two surviving non-transplanted patients (P2 and P19) exhibited atypical and milder clinical courses and survived into adolescence/adulthood. P2 was identified via family screening before symptom onset, and later developed autoimmune and endocrine manifestations, including Hashimoto’s thyroiditis and adrenal insufficiency, which were managed medically. P19 experienced recurrent moderate respiratory infections and extensive cutaneous HPV disease, but no life-threatening infections. Both patients demonstrated low-level residual HLA-DR expression (≈0–4%), with absent or minimal expression on CD45+ lymphocyte and monocytes but detectable expression on activated T cells. After initiation of IgRT and trimethoprim–sulfamethoxazole prophylaxis, both experienced a reduction in infection frequency.

**Figure 2:**
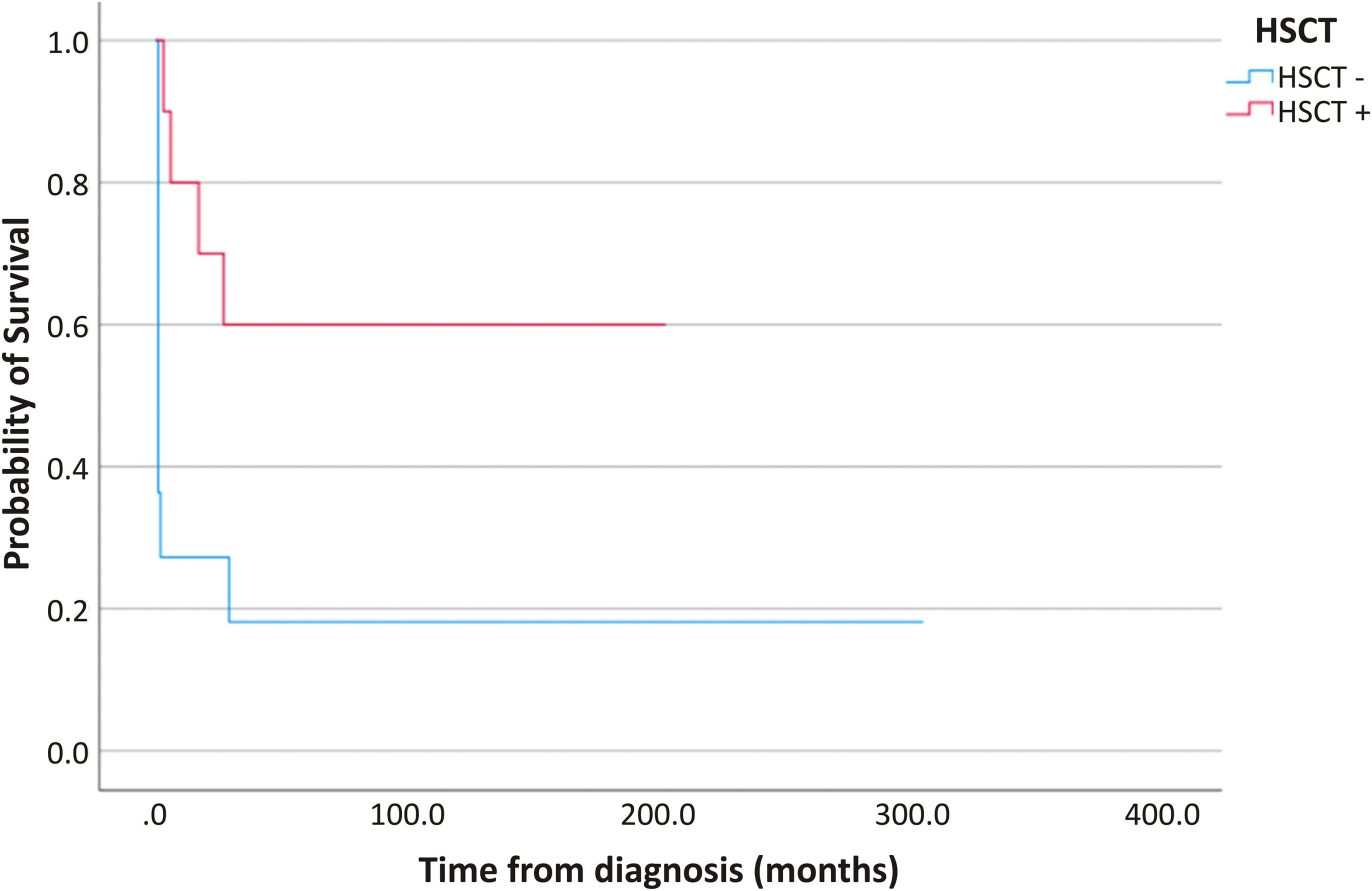
Kaplan–Meier overall survival according to HSCT status (HSCT−, blue; HSCT+, red). Tick marks indicate censored observations. Survival differed significantly between groups (log–rank p = 0.019). All long–term survivors in the HSCT group had follow–up ≥10 years.

Overall survival at last follow-up was 36.4% (8/22). Survival differed markedly according to transplantation status: among patients who underwent HSCT at our center (n=10), six survived (OS 60%), whereas survival among non-transplanted patients was 18% (2/11), limited to individuals with late-onset and atypical clinical phenotypes (**Figure 2**).

## Discussion

In this cohort of 22 patients with MHC class II deficiency, we evaluated the clinical and immunological spectrum, genetic findings, and HSCT outcomes. Consistent with previous reports, most patients presented in early infancy with severe viral pneumonias, opportunistic infections, chronic diarrhea, failure to thrive, and high early mortality (4–19). Although MHC class II deficiency is among CID according to International Union of Immunological Societies (IUIS) criteria (38), its early clinical presentation can closely mimic that of SCID.

In our cohort, after excluding patients with atypical and mild presentations, the median age at symptom onset and diagnosis in MHC class II deficiency was 2.8 months (range, 0.3–6.5 months), and 10 months (range, 0.3–48) respectively, which were comparable to those reported in classical SCID (39). Despite this early onset, the age at diagnosis remained later than in our previously reported SCID cohort, indicating a clinically meaningful diagnostic delay (39). This delay is likely attributable to the preservation of total lymphocyte counts in a substantial proportion of patients, together with the lack of routine assessment of HLA-DR expression in first-line immunological evaluations, which may mask early recognition of this life-threatening disorder.

Delayed diagnosis and advanced disease at presentation were strongly associated with severe infectious complications, intensive care unit admission, and increased mortality. Several infants developed rapidly progressive viral and opportunistic infections leading to critical manifestations, including encephalopathy and acute respiratory distress syndrome, resulting in early death, particularly among those requiring PICU admission at initial presentation. This fulminant course frequently precluded timely referral for HSCT, risking the opportunity for curative treatment. Consistent with previous studies, advanced disease at presentation remains a major determinant of poor outcome in MHC class II deficiency (25–30).

Although the classical early-onset, severe phenotype predominated, our cohort also included a small subset of patients with atypical and relatively milder clinical courses, consistent with previous reports describing long-term survival into adolescence or adulthood without HSCT (1, 2, 7, 21). Notably, these patients demonstrated detectable low-level residual HLA-DR expression, which may partially explain their attenuated clinical phenotype. One patient was diagnosed through family screening while asymptomatic and subsequently developed autoimmune manifestations. Another patient, despite early symptom onset, was diagnosed late and experienced recurrent but non–life-threatening infections, intermittent diarrhea, growth failure, and extensive HPV-associated cutaneous disease.

Compared with large North African cohorts, our series showed a lower prevalence of chronic diarrhea but a higher burden of respiratory infections, which may reflect differences in environmental exposure, referral patterns, and timing of diagnosis. Hepatobiliary complications have been reported relatively frequently in some cohorts of MHC class II deficiency (7–12). However, they were uncommon in our cohort. This may reflect several factors, including reduced *Cryptosporidium* exposure due to improved hygiene and sanitation, early and effective management of viral infections, particularly cytomegalovirus (CMV), and early referral for HSCT, which may have prevented the development of chronic biliary disease. Immune dysregulation was infrequent; autoimmunity was observed in only one patient, which may reflect survival bias or early transplantation (4, 7, 14, 18, 19). Autoimmunity in MHC class II deficiency is thought to result from impaired central and peripheral tolerance due to defective MHC class II expression on thymic epithelial cells, leading to abnormal CD4^+^ T-cell selection and survival of autoreactive clones (14, 40).

Cutaneous eruptions were common in our cohort and frequently occurred during periods of prolonged antimicrobial therapy. Although formal allergy testing was not systematically performed, the clinical pattern predominantly maculopapular rashes occurring during β-lactam or glycopeptide exposure has been associated with delayed, non–IgE-mediated drug reactions in immunodeficient patients rather than true immediate hypersensitivity (14, 41).

Neurological and dysmorphic manifestations are not classical features of MHC class II deficiency but have been increasingly recognized in some cohorts (4, 9, 10, 14, 15, 18, 23). In our series, neurological and ocular findings were heterogeneous, and strabismus was observed follow-up rather than exclusively at diagnosis. These findings suggests that neurodevelopmental and neurological manifestations may be multifactorial, potentially reflecting cumulative infectious burden (particularly CMV), disease course, and treatment-related effects rather than the primary immunodeficiency alone. To explore potential genotype–phenotype associations within our cohort, we compared clinical features across different genetic subgroups. Genetic analysis revealed marked phenotypic heterogeneity with no consistent genotype–phenotype correlation. Several variants identified in this cohort have not been previously reported in public databases (including ClinVar and HGMD) at the time of analysis, further expanding the observed genetic diversity of MHC class II deficiency. Although previous studies have suggested that *RFXANK* mutations may be associated with later symptom onset, and milder disease, and that neurological manifestations have been reported more frequently in patients with *RFXANK* mutations (14, 18), our findings did not support a clear genotype–phenotype association. Patients with RFXANK variants exhibited a broad clinical spectrum, ranging from classical SCID-like disease to relatively milder phenotypes, even within the same family. Among six patients with dysmorphic and/or neurodevelopmental features, genetic data were unavailable in three due to lack of stored DNA samples. In the remaining three patients, variants were identified in RFXAP (n=2) and CIITA (n=1), rather than RFXANK. Overall, neurological manifestations were rare and distributed across different genetic subgroups, further suggesting the notion that these features may not be solely genotype-driven.

However, genetic data were available in only a subset of patients (15/22), which represents a limitation and may restrict the interpretation of genotype–phenotype correlations.

A multicenter study from Türkiye reported recurrent detection of the homozygous *RFX5* c.616G>C missense variant and suggested a possible founder effect within the Turkish population (18). The identification of the same variant in one patient in our cohort is compatible with previous reports suggesting regional enrichment of this mutation; however, confirmation of a founder effect will require larger multicenter studies.

In addition to genotype, residual MHC class II expression has been proposed as a potential modifier of disease severity. In line with previous reports, patients in our cohort with low-level residual HLA-DR expression detected on activated T cells exhibited a comparatively milder clinical course (9, 14, 18), further highlighting the heterogeneity of clinical outcomes and the contribution of immunophenotypic factors beyond the underlying genetic defect.

Immunologically, CD4^+^ T-cell lymphopenia was a prominent and consistent finding, accompanied by marked reductions in naïve CD4^+^CD45RA^+^ T cells and RTEs, supporting impaired thymic output as a central pathogenic mechanism. Reduced total lymphocyte and CD3^+^ T-cell counts were also observed in a subset of patients, suggesting that lymphocyte numbers alone may not reliably distinguish MHC class II deficiency from classical SCID in all cases (18, 19, 30). Even patients with normal absolute CD4^+^ T-cell counts experienced severe clinical disease, emphasizing that functional impairment of the CD4^+^ compartment rather than absolute cell numbers alone underlies disease severity (14, 18, 42, 43).

Although newborn screening (NBS) data based on TREC analysis were not available for the entire cohort, immunological evaluation at diagnosis revealed reduced but variable RTE levels, consistent with a spectrum of thymic dysfunction rather than uniform thymic failure. Accordingly, TREC-based NBS may identify only a subset of affected patients, in contrast to the uniform thymic failure typically observed in classical SCID (16, 44–46). In our cohort, reduced RTE levels together with absent or diminished thymic shadow on imaging in a subset of patients further support this concept. Collectively, these findings indicate that while TREC-based NBS may identify some infants with MHC class II deficiency, it cannot be expected to reliably detect all affected individuals.

Lymphocyte activation responses following PHA stimulation were preserved in the majority of patients. In contrast, humoral immune function was profoundly impaired, with hypogammaglobulinemia and absent or non-protective vaccine-specific antibody responses, consistent with defective CD4^+^ T-cell–dependent B-cell help (7, 47). Reduced HLA-ABC expression was observed in 10 of 17 (59%), patients, with values ranging from 60–88%, in line with previous reports (4, 48). This finding has commonly been attributed to secondary factors such as immune activation, severe infection or treatment related effects. In addition, shared transcriptional regulation of MHC class II and class I genes may contribute to this observation. The RFX complex, comprising *RFXANK*, *RFX5*, and *RFXAP* is essential for MHC class II transcription and has also been implicated in the regulation of MHC class I and β2-microglobulin expression. Accordingly, alterations affecting this complex may contribute to reduced HLA-ABC expression in at least a subset of patients (48).

In addition, increased TCR γδ T-cell frequencies were observed in 4 of 16 evaluated patients, including two with milder clinical phenotypes. Whether this finding reflects a compensatory mechanism remains unclear.

HSCT remains the only curative treatment for MHC class II deficiency. Historically, transplant outcomes have been less favorable than those reported in SCID and other combined immunodeficiencies, mainly due to delayed diagnosis, severe infections at the time of transplantation, and increased transplant-related toxicity (25–30). In addition, unlike SCID, patients often retain autologous T lymphocytes with preserved mitogen-induced proliferative capacity, which may impair engraftment, predispose to mixed chimerism, and limit immune reconstitution (28–30).

Our cohort underscores that patients with early-onset, SCID-like disease who do not undergo HSCT in infancy have an extremely poor prognosis, underscoring the narrow therapeutic window for successful transplantation. Importantly, more recent reports indicate that HSCT outcomes have improved substantially over the last decade (29, 30). Advances in donor availability, graft manipulation, conditioning strategies, and supportive care, together with earlier diagnosis and transplantation at a younger age, have been associated with survival rates approaching those observed in other forms of combined immunodeficiency. Overall, these data suggest that, when performed early and under optimized conditions, HSCT can be a safe and effective curative therapy for children with MHC class II deficiency.

Overall transplant survival was 60% in our cohort, with outcomes appearing to improve in more recent years. Although follow-up duration differed between groups (up to 15 years for MAC and up to 10 years for RTC), survival appeared higher among patients receiving RTC after 2015. This observation may reflect reduced regimen-related toxicity and advances in transplant practice and supportive care over time; however, in the absence of formal statistical comparisons, these findings should be interpreted as descriptive and hypothesis generating only. Pre-transplant viral burden emerged as a key determinant of outcome, as fatal transplant courses were frequently preceded by severe or multiple viral infections, particularly CMV reactivation and respiratory viral infections. While complete viral clearance prior to HSCT is often unachievable, these findings underscore the importance of careful timing of transplantation, close virological monitoring, and aggressive pre-emptive antiviral strategies (29, 30).

Post-transplant immune reconstitution was heterogeneous among survivors, with the main limitation involving thymic-dependent CD4^+^ T-cell recovery. Although immune parameters and donor chimerism were generally favorable; reaching to best within the first year post-HSCT, longitudinal follow-up demonstrated a gradual but clinically insignificant decrease in total lymphocyte counts and T-cell subsets over time, particularly CD4^+^ T cells, which appeared more pronounced in recipients of RTC. A late decline in donor chimerism was observed in some long-term MAC recipients, with chimerism decreasing after 15 years from full engraftment to stable mixed levels of approximately 80–90%. This pattern resembles long-term observations reported after HSCT for severe combined immunodeficiency. By contrast, donor chimerism remained largely stable in RTC recipients; however, follow-up beyond 15 years is not yet available in this group. While naïve CD4^+^ T-cell and RTE proportions remained below age-matched reference ranges, absolute CD4^+^ T-cell counts were generally within acceptable levels, and CD8^+^ compartments were relatively preserved. This pattern likely reflects the persistence of defective thymic epithelial function in MHC class II deficiency, as HSCT corrects only the hematopoietic compartment. The detection of TREC in some patients may indicate preserved early thymocyte development; however, it may not fully reflect effective CD4^+^ T-cell selection within the thymic microenvironment.

Importantly, despite these immunologic findings, none of the surviving patients experienced opportunistic or life-threatening infections, suggesting that immune function remained clinically sufficient. B-cell reconstitution was more favorable, allowing discontinuation of IgRT and development of protective vaccine-specific antibody responses in most survivors. Post-HSCT B-cell subset data, available in a subset of patients, suggested mild alterations in specific B-cell compartments, including reduced switched memory and marginal zone B cells in some individuals. However, these abnormalities did not appear to translate into clinically significant humoral immunodeficiency, as none of the patients required immunoglobulin replacement therapy and no increased susceptibility to infections was observed. These findings should be interpreted with caution due to the small sample size and lack of longitudinal data, but suggest that mild alterations in B-cell subsets may not reflect clinically significant humoral immunodeficiency.

In parallel, HLA-DR expression recovered after HSCT, and HLA-ABC expression normalized at last follow-up.

Importantly, incomplete immune reconstitution and ongoing viral susceptibility may contribute to post-transplant morbidity. In our cohort, neurodevelopmental and sensory sequelae including strabismus, sensorineural hearing loss, and persistent motor or cognitive impairment, either emerged or became more clinically apparent following HSCT, frequently in the context of severe or reactivated viral infections, particularly CMV-related disease, as well as conditioning-related toxicity. Nevertheless, most long-term survivors achieved clinical stability, with a substantially reduced infectious burden and functional humoral immunity, highlighting the potential for meaningful long-term benefit when transplantation is successful.

In contrast, outcomes in non-transplanted patients were poor, with transplant-free survival limited to a small minority with milder phenotypes who benefited from supportive care. Most non-transplanted patients died early in the disease course due to rapidly progressive viral infections and multi-organ involvement, often before curative transplantation could be pursued. Notably, the two surviving patients (P2 and P19) demonstrated atypical clinical courses. P2 was identified through family screening and subsequently developed autoimmune and endocrine complications, specifically Hashimoto’s thyroiditis and adrenal insufficiency, which were managed medically. P19 exhibited a relatively milder phenotype and survived into adolescence, highlighting the spectrum of disease severity and the potential for atypical late-onset presentations in MHC class II deficiency.

In summary, MHC class II deficiency is a life-threatening CID that typically presents in early infancy with a SCID-like phenotype, even in the absence of profound lymphopenia. This dissociation between clinical severity and conventional lymphocyte parameters may contribute to delayed recognition and underscores the need for heightened clinical suspicion. Our findings further expand the phenotypic spectrum of the disease, including neurodevelopmental morbidity, sensory sequelae, and a subset of patients with residual HLA-DR expression who may exhibit atypical and relatively milder disease courses. No consistent genotype–phenotype associations were observed, highlighting the marked clinical and immunological heterogeneity of the disease.

HSCT remains the only curative treatment and was associated with durable engraftment and sustained clinical improvement in our cohort. Outcomes are likely influenced by multiple factors, including timing of diagnosis, pre-transplant infectious burden, and conditioning strategy. While outcomes appeared better in patients receiving RTC, this likely reflects earlier diagnosis and advances in transplant strategies, as these patients were transplanted at younger ages and in more recent years.

Consistent with recent reports, outcomes in our cohort have improved over time. HSCT restored MHC class II expression and resulted in substantial clinical improvement; however, thymic-dependent CD4^+^ T-cell recovery remained variable. Importantly, long-term survivors remained clinically stable without severe opportunistic infections, even in the presence of mixed donor chimerism.

Non-transplanted patients had poor outcomes, whereas HSCT provided effective and long-lasting disease control across conditioning approaches, with no evidence of late graft loss. Early incorporation of HLA-DR analysis into first-line immunological evaluation, prompt referral for transplantation, and meticulous infection monitoring are therefore essential to improve survival and long-term outcomes in patients with MHC class II deficiency.

## Data Availability

The original contributions presented in the study are included in the article/**Supplementary Material**. Further inquiries can be directed to the corresponding author.
