# Supplementary material for: Clinical, immunological, treatment characteristics, and outcomes in 22 patients with major histocompatibility complex class II deficiency
Source: Front Immunol. 2026 Apr 22;17:1811502. doi: 10.3389/fimmu.2026.1811502 (PMC13143760; doi:10.3389/fimmu.2026.1811502)
Supplement: Supplementary file 1 [file DataSheet1.docx]

**Supplementary Table 1. Monoclonal antibodies used for flow cytometric analysis of peripheral blood lymphocyte subsets**

| Marker | Fluorochrome | Clone | Manufacturer |
| --- | --- | --- | --- |
| CD45 | KrO | J33 | Beckman Coulter, Marseille, France |
| CD3 | FITC | UCHT1 | Beckman Coulter, Marseille, France |
| CD4 | ECD | SFCI12T4D11 | Beckman Coulter, Marseille, France |
| CD8 | APC-AF750 | B9.11 | Beckman Coulter, Marseille, France |
| CD16/56 | PE | 3G8+N901 | Beckman Coulter, Marseille, France |
| CD45RA | PC7 | 2H4LDH11LD8 | Beckman Coulter, Marseille, France |
| CD197 (CCR7) | PE | G043H7 | Beckman Coulter, Marseille, France |
| CD19 | APC | J3-19 | Beckman Coulter, Marseille, France |
| CD20 | APC-A750 | B9E9 (HRC20) | Beckman Coulter, Marseille, France |
| CD45RO | PE | UCHL1 | Beckman Coulter, Marseille, France |
| HLA-DR | PB | Immu-657 | Beckman Coulter, Marseille, France |
| TCRg/d | PC7 | IMMU510 | Beckman Coulter, Marseille, France |
| HLA-ABC | FITC | B9.12.1 | Beckman Coulter, Marseille, France |
| CD31 | PE | 1F11 | Beckman Coulter, Marseille, France |

**Supplementary Table 2. Definition of T cell subsets**

| T-cell subset | Immunophenotype |
| --- | --- |
| Naïve CD4⁺ T helper cells | CD45⁺CD4⁺CD45RA⁺CCR7⁺ |
| Central memory CD4⁺ T helper cells | CD45⁺CD4⁺CD45RA⁻CCR7⁺ |
| Effector memory CD4⁺ T helper cells | CD45⁺CD4⁺CD45RA⁻CCR7⁻ |
| TEMRA CD4⁺ T helper cells | CD45⁺CD4⁺CD45RA⁺CCR7⁻ |
| Naïve CD8⁺ T cytotoxic cells | CD45⁺CD8⁺CD45RA⁺CCR7⁺ |
| Central memory CD8⁺ T cytotoxic cells | CD45⁺CD8⁺CD45RA⁻CCR7⁺ |
| Effector memory CD8⁺ T cytotoxic cells | CD45⁺CD8⁺CD45RA⁻CCR7⁻ |
| TEMRA CD8⁺ T cytotoxic cells | CD45⁺CD8⁺CD45RA⁺CCR7⁻ |
| B-cell subset | **Immunophenotype** |
| Switched memory B cells | CD19⁺CD27⁺IgD⁻ |
| Marginal Zone B cells | CD19⁺CD27⁺IgD⁺ |
| Naive B cells | CD19⁺CD27⁻IgD⁺ |
| Activated B cells | CD19⁺CD38^low^CD21^low^ |

)

**Supplementary Table 3. Detailed T-cell subsets at last follow-up after HSCT**

| T-cell subset | P7 | P8 | P9 | Age references |
| --- | --- | --- | --- | --- |
| Naïve CD4⁺ T helper cells (CD45⁺CD4⁺CD45RA⁺CCR7⁺) (%) | 17,3 | 17,9 | 17,2 | 40 (14.9–57) |
| Central memory CD4⁺ T helper cells (CD45⁺CD4⁺CD45RA⁻CCR7⁻) (%) | 51,6 | 39,1 | 51 | 25.4 (15.9–34.6) |
| Effector memory CD4⁺ T helper cells (CD45⁺CD4⁺CD45RA⁺CCR7⁻) (%) | 30,4 | 42,1 | 31,5 | 18.4 (8.9–32.5) |
| TEMRA CD4⁺ T helper cells (CD45⁺CD4⁺CD45RA⁺CCR7⁻) (%) | 0,6 | 0,75 | 0,3 | 12.6 (3.9–39.2) |
| Naïve CD8⁺ T cytotoxic cells (CD45⁺CD8⁺CD45RA⁺CCR7⁺) (%) | 49,68 | 45,9 | 51,8 | 26.1 (7–45.3) |
| Central memory CD8⁺ T cytotoxic cells (CD45⁺CD8⁺CD45RA⁻CCR7⁺) (%) | 19 | 23,4 | 10,7 | 2.2 (1.5–3.9) |
| Effector memory CD8⁺ T cytotoxic cells (CD45⁺CD8⁺CD45RA⁻CCR7⁻) (%) | 16,5 | 24,6 | 25,9 | 26.4 (17–42.4) |
| TEMRA CD8⁺ T cytotoxic cells (CD45⁺CD8⁺CD45RA⁺CCR7⁻) (%) | 14,8 | 6,1 | 11,5 | 43.4 (21.8–65.7 |

**Supplementary Table 4. Detailed B-cell subsets at last follow-up after HSCT**

| **B-cell subset** | **P7** | **P8** | **P9** | **P13** | **P15** | **Age references** |
| --- | --- | --- | --- | --- | --- | --- |
| **Switched memory B (CD19+ CD27+ IgD-) (%)** | 26,2 | 14,7 | 26,1 | 3,3 | 11,9 | 12,9–45 |
| **Marginal Zone B (CD19+ CD27+ IgD+) (%)** | 2,6 | 6,9 | 7 | 4,8 | 4,8 | 5,1–11,8 |
| **Naive B (CD19+CD 27- IgD+) (%)** | 38,8 | 67,6 | 41 | 86 | 70 | 62,2–76,2 |
| **Activated B (CD19+ CD38 ^low^ CD21^low^) (%)** | 2 | 2,1 | 1 | 4,3 | 14,4 | 3,2–5,9 |
